# A Pathological Image-Based Pathomics Signature Combined with Clinicopathological Features for Predicting Lymph Node Metastasis in Esophageal Squamous Cell Carcinoma: A Development and External Validation Study

**DOI:** 10.3390/cancers18203248

**Published:** 2026-10-09

**Authors:** Kaiming Peng, Jieming Lu, Peipei Zhang, Weiguang Zhang, Jinlong Fang, Junhuang Lin, Shuchen Chen, Mingqiang Kang

**Affiliations:** 1 Department of Thoracic Surgery, Fujian Medical University Union Hospital, Fuzhou 350001, China; drpengkm@163.com (K.P.); jameslou2068@163.com (J.L.); zhangpeipei0591@163.com (P.Z.); zwgitsachance@126.com (W.Z.); fangjinlong0312@163.com (J.F.); 18876331642@163.com (J.L.); cscdoctor@163.com (S.C.); 2 Key Laboratory of Gastrointestinal Cancer, Ministry of Education, Fujian Medical University, Fuzhou 350001, China; 3 Fujian Key Laboratory of Tumor Microbiology, Department of Medical Microbiology, Fujian Medical University, Fuzhou 350001, China; 4 Key Laboratory of Cardio-Thoracic Surgery, Fujian Medical University, Fuzhou 350001, China; 5 Clinical Research Center for Thoracic Tumors of Fujian Province, Fuzhou 350001, China

**Keywords:** esophageal squamous cell carcinoma, lymph node metastasis, pathomics, nomogram, machine learning, prognosis

## Abstract

**Background**: Esophageal squamous cell carcinoma (ESCC) accounts for over 80% of esophageal cancer cases globally. Lymph node status is the strongest determinant of survival after resection, yet occult disease is found in about 17% of patients classified as node-negative. We developed a postoperative model estimating the probability of lymph node metastasis (LNM) from the resection specimen, combining nuclear features with clinicopathological variables. **Methods**: Of 477 consecutive ESCC patients undergoing radical esophagectomy without neoadjuvant therapy, 416 were analyzed (LNM 217, 52.2%). Nuclear features were extracted from H&E whole-slide images (20×) by QuPath optical-density detection; LASSO logistic regression selected 17. A nomogram combining the pathomics score with age, sex, tumor location, pT category and grade was validated by 10-fold cross-validation (out-of-fold, OOF) and applied without refitting to 101 patients from another institution. **Results**: OOF AUC was 0.821 (95% CI 0.779–0.858) for the combined model, versus 0.791 (clinical) and 0.762 (pathomics alone). The pathomics score remained an independent predictor (adjusted OR 2.47, 95% CI 1.80–3.39; *p* < 0.001). LNM prevalence rose across tertiles (15.8%, 55.1%, 85.6%; *p* < 0.001). Among 199 node-negative patients, high pathomics risk predicted worse 3-year overall survival (77.2% vs. 94.3%; *p* = 0.005) and progression-free survival (75.5% vs. 88.9%; *p* = 0.007). Externally (*n* = 101; LNM 24.8%), AUCs were 0.812, 0.801 and 0.697, and adding pathomics did not significantly improve discrimination. **Conclusions**: Within the development cohort, pathomics added modest but significant incremental value and identified node-negative patients at elevated risk. Because the model requires the resection specimen, it cannot guide preoperative treatment selection. External validation did not confirm incremental value: the clinical component; the pathomics signature did not.

## Simple Summary

Esophageal cancer is a leading cause of cancer death, and whether the tumor has spread to the lymph nodes is the strongest factor determining survival. After the tumor is removed, pathologists look for lymph-node spread in the tissue they receive, but involved nodes can be missed, and some patients reported as node-negative go on to relapse. We used a computer method to measure nuclear shape and staining on ordinary microscope slides of the removed tumor, and combined those measurements with routine patient information to estimate each patient’s risk of lymph-node involvement. In 416 patients from our hospital, the combined approach estimated nodal involvement better than routine factors alone and flagged node-negative patients at higher risk of later recurrence or death. Tested unchanged on 101 patients from another hospital, the patient-information part still worked while the slide part did not. Shared scanning and staining standards are needed before wider use.

## 1. Introduction

Esophageal squamous cell carcinoma (ESCC) remains a leading cause of cancer-related mortality worldwide, particularly in East Asia and sub-Saharan Africa [1,2]. In 2022, esophageal cancer ranked eleventh in global incidence and seventh in cancer mortality, with an estimated 511,054 new cases and 445,391 deaths [1]. Despite advances in multimodal therapy, lymph node metastasis (LNM) is one of the strongest prognostic factors determining survival after surgical resection [3]. In patients staged as node-negative by conventional imaging and endoscopic ultrasound, occult nodal disease is far from rare: immunohistochemical re-examination of 516 completely resected pN0 ESCC specimens identified occult lymph node metastases in 17.1%, and those patients had 5-year overall and disease-free survival of only 53.9% and 50.5% (comparable to pN1 patients), with occult disease remaining an independent prognostic factor [4].

Before surgery, nodal involvement is assessed by computed tomography (CT), positron emission tomography (PET), and endoscopic ultrasonography (EUS), which have limited sensitivity for micrometastases and small-volume nodal involvement [5,6]. After resection, the pathologist assigns nodal status from the nodes that were retrieved, and that assessment depends on how many nodes are recovered and how thoroughly they are examined; a yield below the conventional 12-node threshold is considered inadequate for reliable nodal staging. The TNM staging system, while universally adopted, does not incorporate quantitative histomorphological information from the primary tumor that may reflect its biological aggressiveness and metastatic propensity [7].

Pathomics, also termed computational pathology or histomics, refers to the high-throughput extraction of quantitative morphological, intensity, and texture features from digitized histopathological images [8,9]. By converting H&E-stained tissue sections into mineable, standardized quantitative data, pathomics can capture subtle patterns of nuclear pleomorphism, spatial tissue architecture, and staining heterogeneity that escape visual assessment [8,9], and enables reproducible, benchmarked feature extraction [10]. Recent studies have demonstrated the feasibility of pathomic signatures for predicting treatment response and prognosis [11], as well as molecular alterations [12,13], in esophageal and other gastrointestinal cancers, and deep learning on H&E whole-slide images has identified cancerous and precancerous esophagus tissue [14] and predicted nodal metastasis from lymph-node sections in gastric cancer [15]. However, the application of pathomics to predict LNM specifically in ESCC remains underexplored.

In this study, we developed a pathomics signature from H&E-stained whole-slide images of the resected primary ESCC tumor and combined it with established clinicopathological predictors (age, sex, tumor location, pathological T category, and histological grade) in a postoperative nomogram. Our primary objective was to evaluate whether quantitative pathomic features provide independent and incremental value beyond these conventional clinicopathological factors for estimating the probability of pathological lymph node involvement. As a secondary, hypothesis-generating objective, we assessed whether the pathomics score could identify, among patients classified as node-negative by standard pathology, a subgroup at higher risk of recurrence or death.

## 2. Materials and Methods

This study was conducted and reported in accordance with the STROBE statement [16] (Strengthening the Reporting of Observational Studies in Epidemiology) and the TRIPOD+AI statement [17] (Transparent Reporting of a multivariable prediction model for Individual Prognosis Or Diagnosis, extended to Artificial Intelligence). It also aligns with the REMARK reporting guidelines for tumor-marker prognostic studies [18].

### 2.1. Study Design and Patients

This retrospective study was conducted at Fujian Medical University Union Hospital and approved by the Institutional Review Board (approval no. 2024KY060). The requirement for informed consent was waived due to the retrospective nature of the study.

**Figure 1:**
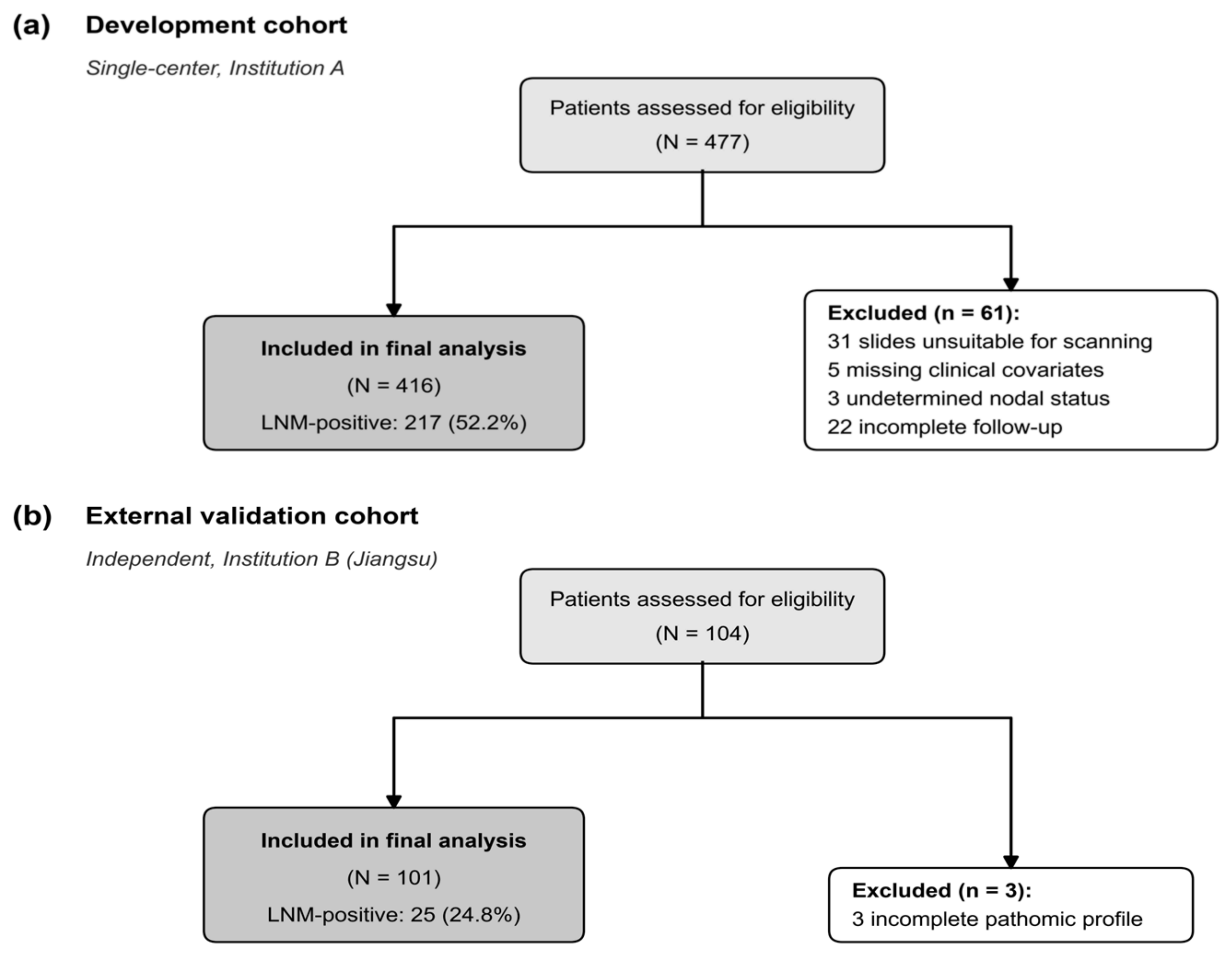
STROBE flow diagram of patient enrolment and cohort partitioning. (**a**) Development cohort (single center, Institution A): patients who had received neoadjuvant therapy were excluded before enrolment; 477 consecutive eligible patients were enrolled, and 61 were then excluded (31 with slides unavailable or unsuitable for digital scanning, 5 with missing clinical covariates, 3 with undetermined nodal status, and 22 with incomplete follow-up); 416 patients were included in the final analysis (pN0, 199; pN+, 217). (**b**) Independent external validation cohort (Institution B, Jiangsu): 104 patients assessed, 3 excluded because a complete pathomic feature profile could not be extracted, and 101 included.

We consecutively enrolled 477 patients with histologically confirmed ESCC who underwent radical esophagectomy between 2012 and 2017. Inclusion criteria were: (1) primary ESCC confirmed by preoperative biopsy; (2) R0 resection achieved; (3) availability of H&E-stained tumor slides suitable for digital scanning; and (4) complete clinicopathological and follow-up data. All 477 enrolled patients had undergone upfront surgery: those who had received neoadjuvant therapy were excluded before enrollment and are therefore not represented in this series, because neoadjuvant treatment alters both components of the model. It induces tumor regression with fibrosis, necrosis, and treatment-related nuclear changes that compromise the morphometric features measured on H&E slides, and it converts the outcome into a treatment-response phenotype (ypN) rather than a measure of the tumor’s intrinsic propensity for nodal spread. Nodal status can, of course, still be assessed histologically after neoadjuvant therapy; it is the interpretability of the predictor outcome pair, not the feasibility of nodal assessment, that motivated this exclusion. Of the 477 enrolled patients, 61 were excluded during central image-quality review and data verification: 31 because the H&E slides proved unavailable or unsuitable for digital scanning, 5 because of missing clinical covariates, 3 because nodal status could not be determined from the pathology record, and 22 because of incomplete follow-up. The final cohort comprised 416 patients (Figure 1). Multiple imputation was not employed: the 61 exclusions represented 12.8% of enrolled patients, missingness was concentrated in slide availability and follow-up completeness rather than in scattered covariates, and the complete-case analytical sample was large enough for stable model fitting.

### 2.2. Pathological Image Acquisition and Feature Extraction

Formalin-fixed paraffin-embedded (FFPE) tumor blocks were sectioned at 4 μm thickness and stained with H&E using a standardized protocol. Representative sections in which the invasive tumor component occupied a large proportion of the tissue were selected by an experienced pathologist who was blinded to lymph node status and survival outcomes; slide quality was assessed by the same pathologist, and sections of insufficient quality or with inadequate tumor content were excluded. Selection was performed centrally as part of retrospective data assembly, and the selections were reviewed by a second pathologist, who was blinded in the same way. Whole-slide images (WSIs) were acquired using a KFBIO KF-PRO-400-HI digital slide scanner (KFBIO, Ningbo, China) at 20× optical magnification (pixel resolution 0.5 μm/pixel).

Image analysis was performed using QuPath (version 0.5.1), an open-source platform for digital pathology image analysis [19]. Nuclei were detected automatically using the hematoxylin optical density (OD) algorithm with the following parameters: pixel size 0.5 μm; background radius 8 μm; sigma 1.5 μm; OD threshold 0.1; maximum background intensity 2.0; remaining parameters at default values. No tumor region of interest was delineated: the entire selected section was analyzed, and non-neoplastic elements present within the analyzed area, including stromal and inflammatory cells, were therefore included in feature computation. Each WSI was partitioned into tiles using QuPath’s built-in tiling function, and nuclei were detected within each tile; across the analyzed section, nuclei were detected on the order of 10^5^ to 10^6^ per case (tumor and non-neoplastic nuclei combined). No case that entered image analysis failed feature extraction. For each tile, 41 pathomic features were extracted, encompassing nuclear morphology (area, perimeter, circularity, eccentricity, caliper dimensions), hematoxylin optical density (OD) metrics (sum, mean, standard deviation, minimum, maximum, range), eosin OD metrics (sum, mean, standard deviation, minimum, maximum, range), cytoplasmic OD metrics, and the nucleus-to-cell area ratio. For each feature, the per-nucleus measurements obtained across the analyzed section were aggregated to a single patient-level value by computing the standard deviation across all detected nuclei.

### 2.3. Clinical and Pathological Variables

The following clinicopathological variables were obtained from the medical record and the postoperative pathology report: age at surgery (continuous, years); sex (male/female); tumor location (upper/middle/lower thoracic esophagus); pathological T category (pT); histological grade of the resected tumor (G1 well-differentiated, G2 moderately differentiated, G3 poorly differentiated/undifferentiated); and total number of lymph nodes retrieved. Carcinoma in situ (Tis; *n* = 11) was pooled with pT1 for descriptive purposes only, and in Table 1 pT is collapsed to pT1/pT2 versus pT3/pT4 for descriptive comparison. For modeling, pT was entered as a single ordinal term in which Tis was coded 0.5, half a category below pT1, because Tis lacks invasion of the lamina propria and is expected to carry a lower risk of nodal involvement than pT1; a separate coefficient was not estimated for a group of this size. The primary endpoint was postoperative LNM status (pN0 vs. pN+) determined by histopathological examination according to the AJCC/UICC TNM classification system (8th edition). Secondary endpoints included overall survival (OS, defined as time from surgery to death from any cause) and progression-free survival (PFS, defined as time from surgery to first documented recurrence or death). Every predictor in the model is derived from the resection specimen or from the pathology report that accompanies it. The model is therefore a postoperative pathological model for estimating the probability of nodal involvement once the resection specimen has been reported, and it is not designed to inform preoperative treatment selection.

### 2.4. External Validation Cohort and Procedures

To assess transportability beyond the development center, the frozen combined nomogram, including the StandardScaler transform, the LASSO-selected pathomics weights and the logistic regression coefficients, was applied without any refitting to an independent external cohort of 101 ESCC patients treated at a separate institution (Jiangsu); these patients were enrolled consecutively between 2011 and 2012 and 25 (24.8%) had pathologically confirmed LNM. Eligibility criteria were identical to those used in the development cohort, and LNM status was determined by the same postoperative histopathological assessment (AJCC/UICC 8th edition). Because this cohort was assembled from archival material, the only additional exclusions were three patients whose slides did not yield a complete 41-feature profile from the automated QuPath pipeline (104 patients assessed; 3 excluded; 101 analyzed). The use of archival specimens and records from both centers was covered by the institutional review board approval obtained for the parent study (approval no. 2024KY060; see Institutional Review Board Statement). Pathomic features were extracted from the external H&E whole-slide images using the identical QuPath pipeline and the same 41-feature set, with no region of interest delineated at either center, and clinical variables were mapped onto the same schema used in the development cohort. At both centers, slide scanning and H&E staining followed the same protocol (identical scanner model and section thickness of 4 μm), whereas tissue fixation and processing followed each center’s routine archival practice and were not prospectively harmonized. Because pre-analytical handling can influence nuclear optical-density measurements, we evaluated, and in exploratory analyses attempted, batch-effect correction (ComBat) [20]; it did not improve external performance and was therefore not adopted, so all external results reflect the uncalibrated frozen model. We summarized external discrimination by the AUC, with 95% confidence intervals from 2000 bootstrap resamples, and calibration by the slope, intercept, and Brier score. We compared models with DeLong’s test and, as a sensitivity analysis, with paired bootstrap resampling (5000 replicates, stratified by event status). Because the external predicted probabilities were highly concentrated near the top of the range (median 0.962, interquartile range 0.923–0.984) and the candidate models are nested, DeLong’s parametric covariance estimator can be unstable; where the two tests disagreed, we report the bootstrap *p*-value. We stratified risk by tertiles of the nomogram-predicted probability and report two definitions of the risk groups. First, because the nomogram was applied externally as a frozen model without recalibration, tertiles were derived within each cohort separately, so that the cutoffs match each cohort’s own predicted-probability distribution. Second, we also applied the single set of cutoffs derived in the development cohort (0.335 and 0.741) to the external cohort without modification, so that one set of fixed cutoffs is used across both cohorts. We tested both risk gradients for trend with the Cochran –Armitage test.

The two cohorts differed in several respects: patients in the external cohort were older (63.6 ± 7.2 vs. 58.5 ± 8.3 years), more often had middle-third tumors (81.2% vs. 61.5%), and had a markedly lower prevalence of lymph node metastasis (24.8% vs. 52.2%). Because the frozen model was applied without recalibration, these case-mix differences are a plausible contributor to the lower external discrimination and should be considered when interpreting the estimates.

### 2.5. Model Development and Statistical Analysis

Internal validation used a nested cross-validation scheme. The cohort was partitioned into ten stratified folds; within each fold, feature standardization, LASSO feature selection and logistic coefficient estimation were repeated from scratch using only the nine training folds, so that every patient’s out-of-fold (OOF) prediction was produced by a pipeline in which that patient contributed to neither feature selection nor model fitting. All performance estimates reported below (discrimination, calibration, Brier score, decision curve analysis and model comparisons using the DeLong test, NRI and IDI) were computed on these OOF predictions. The nomogram and the risk groups and survival strata derived from it use the coefficients fitted once on all 416 patients, because risk groups intended for use must be defined by a single deployable model rather than by ten different fold-specific models.These grouped analyses are therefore apparent (resubstitution) estimates by design, and we report them as exploratory, to be read alongside the OOF performance estimates.

Feature selection was performed using LASSO (Least Absolute Shrinkage and Selection Operator) logistic regression with 5-fold cross-validation to identify the optimal penalization parameter (λ) minimizing the deviance. Features with non-zero coefficients at the optimal λ were retained, and their linear combination constituted the pathomics score (pathScore).

Three logistic regression models were developed and compared:

1. Clinical model: sex, age, tumor location, T category, histological grade.
2. Pathomics model: pathScore alone.
3. Combined model: all clinical variables plus pathScore.

Discrimination was assessed by the area under the receiver operating characteristic curve (AUC). For internal validation, OOF AUCs with 95% confidence intervals (bootstrap, 2000 resamples) were computed from the out-of-fold predictions; apparent (resubstitution) AUCs, obtained by refitting each model on the full cohort, were reported alongside to quantify optimism (apparent minus OOF). Pairwise comparison of models was performed using DeLong’s test [21]. These intervals resample the out-of-fold predictions and therefore hold the selected feature set fixed. As a sensitivity analysis we repeated the procedure with 2000 bootstrap replicates in which the whole development process was rerun inside each replicate: standardization, LASSO selection and coefficient estimation were refitted from scratch in ten nested folds on the resampled patients only, and out-of-fold predictions were taken from that replicate’s own pipeline. The interval for the incremental ΔAUC of the combined model over the clinical model then moved away from the null rather than towards it (95% CI 0.011 to 0.074, bootstrap *p* = 0.003, against 0.003 to 0.055 for the interval), while the interval for the comparison with the pathomics-only model was essentially unchanged (0.025 to 0.089 against 0.025 to 0.092); the number of LASSO-selected features varied across replicates (median 15, interquartile range 14 to 17, range 9 to 21, against 17 in the full cohort). Both sets of intervals and the feature-count distribution are reported in Table S5. We retained the intervals obtained from the out-of-fold predictions as the primary result, because bootstrap replicates contain duplicated observations, which makes the out-of-fold estimates within a replicate optimistic (mean combined AUC 0.832 across replicates against the out-of-fold value of 0.821) and therefore favors the model with more parameters. Model calibration was assessed graphically by calibration plots and quantitatively by Brier scores. The net clinical benefit of each model was evaluated using decision curve analysis (DCA) [22].

The independent prognostic value of the pathomics score was tested by incorporating it into a multivariable logistic regression alongside clinical factors, reporting odds ratios (ORs) with 95% confidence intervals and Wald *p*-values. Incremental value was assessed by likelihood-ratio tests comparing nested models.

For risk stratification, patients were categorized into low-, intermediate-, and high-risk groups based on tertiles of the combined nomogram-predicted probability. The Cochran–Armitage test for trend was used to assess the monotonic relationship between risk group and LNM prevalence.

Secondary survival analyses were conducted: (1) within the pN0 subgroup, Kaplan–Meier curves and log-rank tests compared OS and PFS between patients dichotomized by the median pathScore within the pN0 subgroup (an exploratory, data-driven cutoff defined as the subgroup median); univariate Cox proportional-hazards models reported hazard ratios (HRs) per 1-SD increase in continuous pathScore; and (2) across the entire cohort, Kaplan–Meier curves compared OS and PFS among the three nomogram-derived risk groups.

All statistical tests were two-sided, and *p* < 0.05 was considered statistically significant. Analyses were performed using Python (version 3.11.x) with scikit-learn, statsmodels, lifelines, and matplotlib.

As a sensitivity analysis for overall survival, we updated vital status against an institutional esophagectomy database covering 2010–2020. We matched 396 of 416 patients (95.2%) by medical record number. Deaths recorded in either source were counted as events, and follow-up time was extended for patients still alive at the later database lock. The node-negative analyses were then repeated. Progression-free survival was not updated, because the extended source recorded recurrence dates only rather than progression-free survival, and mixing the two definitions would change the endpoint.

## 3. Results

### 3.1. Patient Characteristics

Of 477 enrolled patients, 416 were included in the final analysis (Figure 1). The mean age was 58.5 years (SD 8.3), and 306 (73.6%) were male. Tumors were located in the middle thoracic esophagus in 256 (61.5%) patients, followed by lower (126, 30.3%) and upper (34, 8.2%) locations. Histological grades were predominantly G1 (174) and G2 (213), with G3 tumors accounting for 29 cases. The median number of retrieved lymph nodes was 30 (IQR 23–38; mean 32.0, SD 12.8, range 7–79), and 407 of 416 patients (97.8%) had at least 12 nodes retrieved. Lymph node dissection followed the same institutional protocol in 397 patients (95.4%), consisting of standard two-field (thoraco-abdominal) dissection; the remaining 19 patients (4.6%) underwent three-field dissection with additional cervical node dissection, and all of them had 22 or more nodes retrieved. Low nodal yields were therefore uncommon and were not attributable to variation in the extent of dissection. Compared with the 61 excluded patients, the included cohort did not differ significantly in age (58.5 vs. 58.2 years, *p* = 0.79), sex (73.6% vs. 75.4% male, *p* = 0.88), histological grade (*p* = 0.94) or tumor location (*p* = 0.10), indicating no material selection bias with respect to these characteristics; T category and nodal status were not compared because they were unavailable or indeterminate in the excluded patients by definition.

LNM was identified in 217 patients (52.2%). Compared with node-negative patients, those with LNM were more likely to be male (79.7% vs. 66.8%), had more advanced T categories, higher-grade tumors, and a slightly greater number of retrieved lymph nodes (33.0 vs. 31.0). Detailed characteristics are summarized in Table 1.

### 3.2. Feature Selection and Pathomics Score Construction

**Figure 2:**
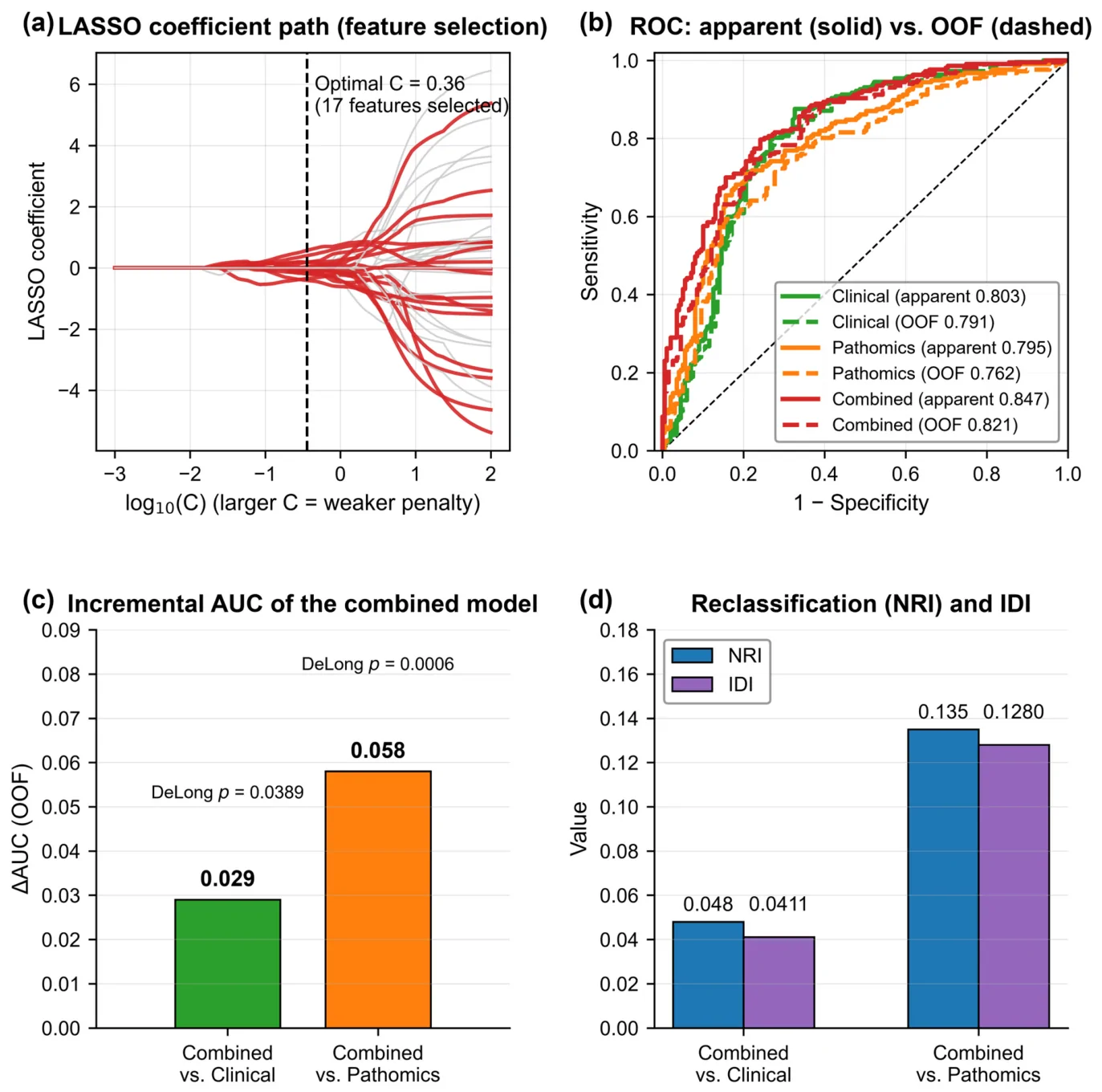
Pathomic feature selection and model discrimination. (**a**) LASSO coefficient regularization path; each line shows the trajectory of one pathomic feature’s coefficient as the penalty relaxes (log10 C increases left→right), and the vertical dashed line marks the optimal penalty selected by 5-fold cross-validation (C = 0.36; in scikit-learn C is the inverse of the regularization strength, C = 1/λ), at which 17 features (red) remain in the model; gray lines denote features shrunk to zero. (**b**) Receiver operating characteristic (ROC) curves of the three models (*N* = 416); solid lines show apparent (resubstitution) estimates and dashed lines show cross-validated out-of-fold (OOF) estimates (combined OOF AUC = 0.821, 95% CI 0.779–0.858). (**c**) Incremental ΔAUC (out-of-fold) of the combined model over single-modality baselines, with DeLong *p*-values. (**d**) Net reclassification improvement (NRI) and integrated discrimination improvement (IDI); bootstrap B = 2000.

From the initial pool of 41 candidate pathomic features, LASSO logistic regression selected 17 non-zero features (Figure 2a) associated with LNM status when fitted on the full cohort (the apparent framework). The selected features predominantly captured nuclear shape irregularity (eccentricity, caliper dimensions), hematoxylin OD variation (reflecting chromatin density heterogeneity), and eosin OD distribution patterns (reflecting cytoplasmic texture). These features were linearly weighted by their LASSO coefficients to form a composite pathomics score (pathScore), which was then standardized (mean = 0, SD = 1) before incorporation into the combined model. Consistent with the prespecified analytic plan, feature selection was performed on the full cohort; the resulting apparent estimates are shown only to illustrate the selected signature, whereas all performance and incremental-value conclusions in this study are based on cross-validated out-of-fold (OOF) predictions.

### 3.3. Multivariable Analysis and Model Performance

In multivariable logistic regression (Table 2), the pathomics score was independently associated with LNM (adjusted OR per SD 2.47, 95% CI 1.80–3.39; *p* < 0.001), an effect size comparable to that of pT category (OR per step 2.62, 95% CI 1.90–3.60; *p* < 0.001); the two confidence intervals overlap almost completely. Histological grade was also significant (OR 1.68, 95% CI 1.12–2.52; *p* = 0.013). Sex, age, and tumor location did not reach statistical significance. The addition of the pathomics score to the clinical model yielded significant improvement in fit (likelihood-ratio χ^2^ = 35.5; *p* = 2.56 × 10^−9^).

Variable labels are presented in reader-friendly form. Odds ratios are reported per unit increase in each predictor as entered into the model: per year for age; male versus female for sex; per one-stage increment for pT; per one-grade step for histological grade; per one-step increase in tumor location (upper→middle→lower); and per 1 SD of the pathomics score (a z-standardized LASSO-derived linear predictor). Continuous predictors were entered on their native scales.

**Figure 3:**
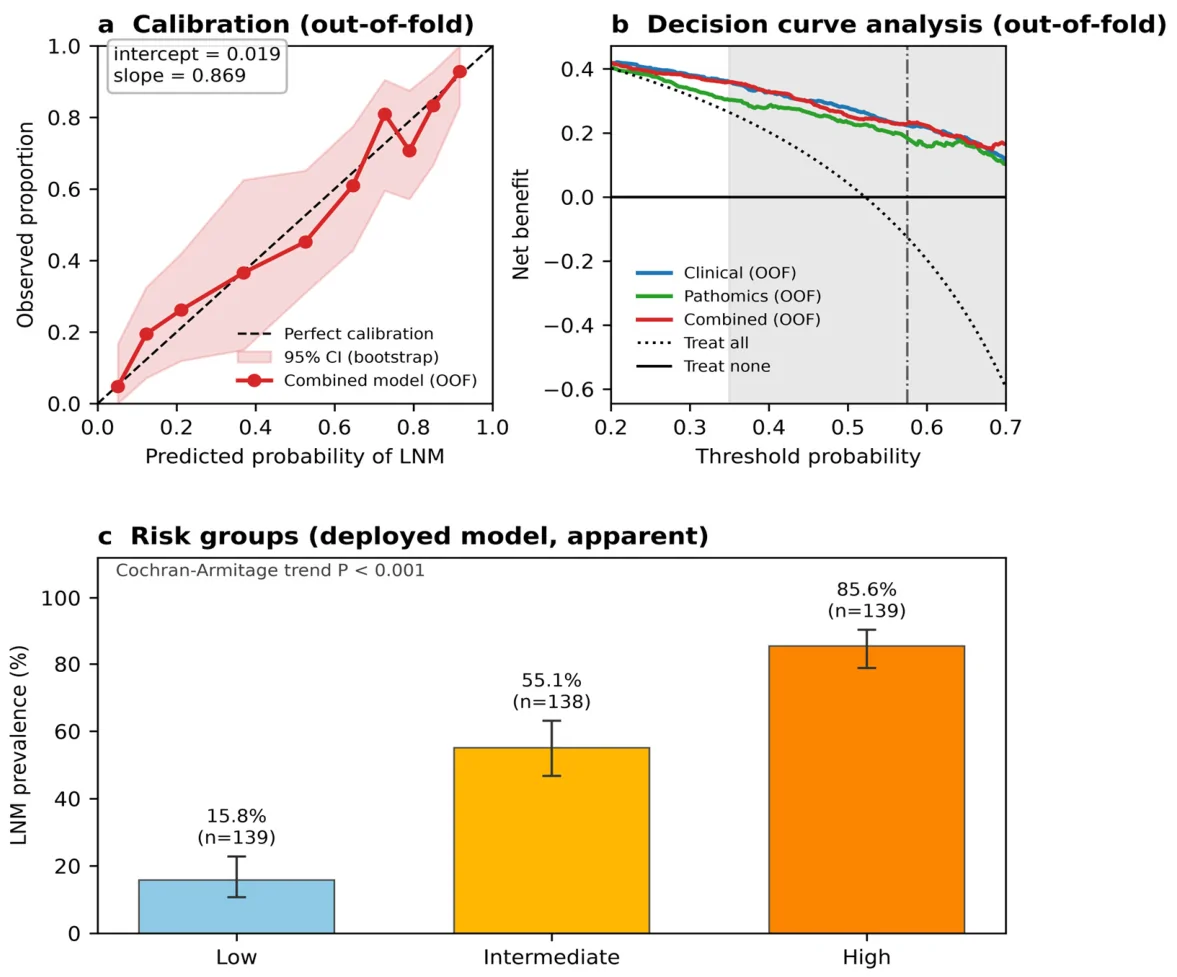
**Clinical utility of the combined nomogram. (a,b) Based on out-of-fold predictions.** (**a**) Calibration plot; the diagonal dashed line represents perfect calibration and the shaded band is the 95% confidence interval from 2000 bootstrap resamples. Calibration intercept (calibration-in-the-large) = 0.019 and slope = 0.869, indicating that predicted probabilities are close to ideal (intercept ≈ 0, slope ≈ 1). (**b**) Decision curve analysis, with treat-all and treat-none strategies shown as references. Because the model is applied after resection, when nodal status is already known, its only plausible clinical use is in patients classified pN0, in whom the question is whether the probability of occult nodal involvement is high enough to justify intensified surveillance or consideration of adjuvant therapy; the displayed threshold range (20% to 70%) was chosen to span the probabilities assigned within the pN0 subgroup, whose high-pathomics-risk stratum (Figure 5a,b) had a median out-of-fold predicted probability of 0.58 (IQR 0.35–0.73). The management consequences implied by these thresholds generate, but do not establish, a hypothesis that requires prospective testing. (**c**) LNM prevalence across risk groups defined by the probabilities of the deployed nomogram fitted on the full cohort (low 15.8%, intermediate 55.1%, high 85.6%; Cochran–Armitage trend *p* < 0.001); these grouped estimates are apparent by design (Section 2.5) and are presented as exploratory.

Across 10-fold cross-validation (Table 3), the combined model achieved an out-of-fold AUC of 0.821 (95% CI 0.779–0.858), which was significantly superior to the pathomics-only model (AUC 0.762, 95% CI 0.715–0.806; DeLong *p* = 0.0006) and also to the clinical model, although the margin was smaller (AUC 0.791, 95% CI 0.744–0.835; *p* = 0.039). The apparent (resubstitution) AUC of the combined model was 0.847, indicating a modest optimism of +0.026 that was mitigated by cross-validation. The Brier score for the combined model was 0.173, and the calibration slope and intercept were 0.869 and 0.019, respectively, indicating that predicted probabilities were well calibrated (slope near 1, intercept near 0) (Figure 3a).

Because apparent (resubstitution) AUCs are optimistically inflated by refitting the model on the full cohort, all performance and incremental-value conclusions in this manuscript are based on the cross-validated out-of-fold estimates. The apparent (solid) curves in Figure 2b are reported only to convey the magnitude of optimism relative to the OOF (dashed) curves.

To quantify the clinical benefit of integrating pathomics into clinical prediction, we computed the net reclassification improvement (NRI), integrated discrimination improvement (IDI), and ΔAUC (Figure 2c,d), and assessed clinical usefulness by decision curve analysis (Figure 3b). Compared with the clinical model alone, adding pathomics yielded a modest but statistically significant ΔAUC of 0.029 (DeLong *p* = 0.0389), NRI = 0.048 (95% CI −0.039–0.164), and IDI = 0.0411 (95% CI 0.0137–0.0669). In contrast, the improvement over the pathomics-only model was more substantial: ΔAUC = 0.058 (DeLong *p* = 0.0006), NRI = 0.135 (95% CI 0.000–0.235), and IDI = 0.1280 (95% CI 0.0969–0.1579). These findings indicate that clinicopathological and pathomic information are complementary rather than redundant: each contributes independent signal, and their integration, not either modality alone, yields the strongest discrimination(Figure 2). Pathomics thus provides a small but consistent increment to established clinicopathological risk assessment (Figure 3). Repeating the bootstrap with the entire development process rerun inside each of 2000 replicates widened this interval but moved it away from the null (ΔAUC 95% CI 0.011 to 0.074, bootstrap *p* = 0.003; Table S5).

### 3.4. Risk Stratification

**Figure 4:**
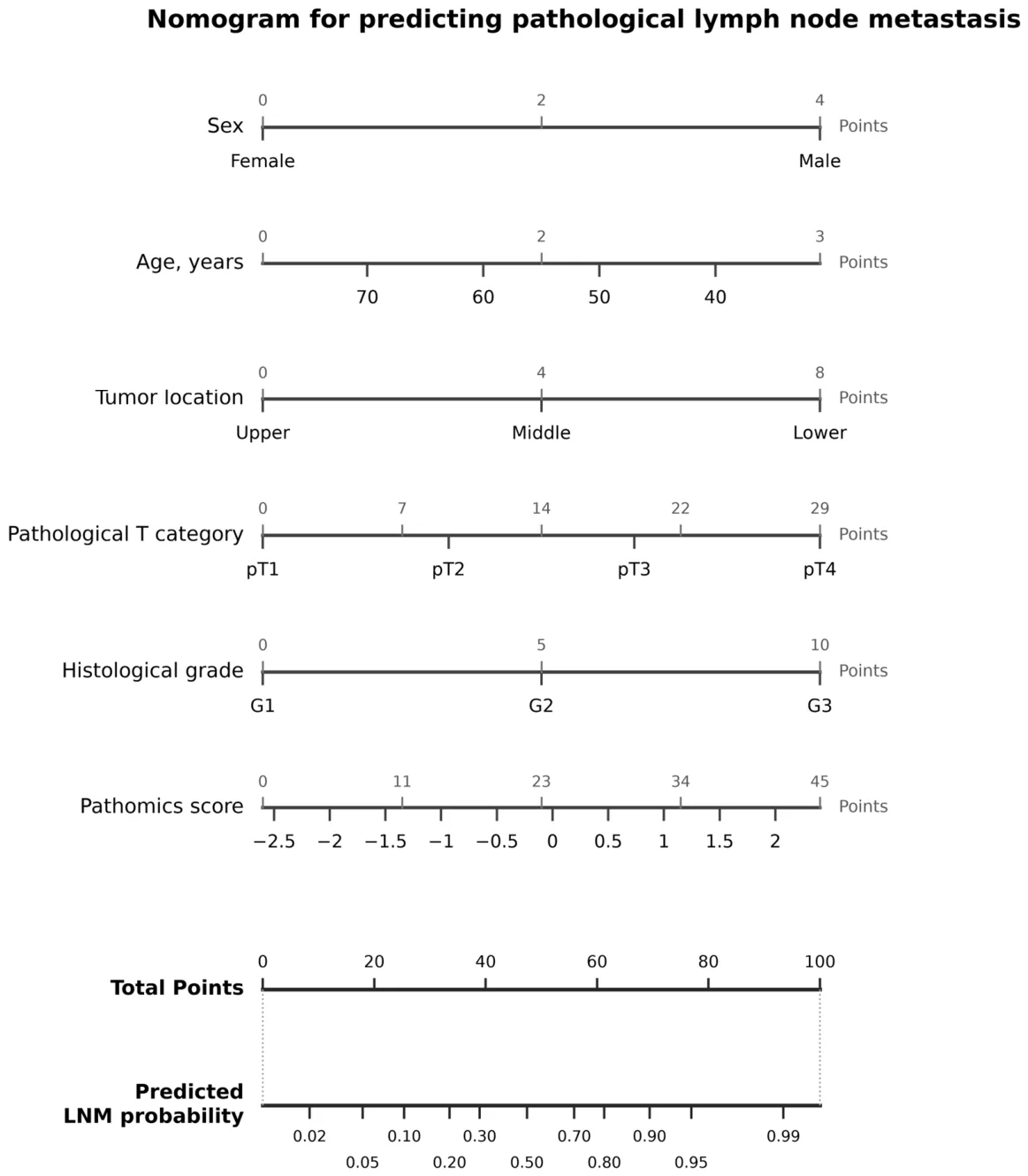
**Postoperative nomogram combining clinicopathological variables and the pathomics score to estimate the probability of pathological LNM.** Every axis is graduated in clinically selectable categories: sex (female/male), age (years), tumor location (upper/middle/lower thoracic), pathological T category (pT1–pT4; Tis is not shown as a separate tick and should be read as about 5 points below pT1, corresponding to an odds ratio of 0.62 relative to pT1; see Section 2.3), histological grade (G1/G2/G3) and the continuous pathomics score. To use: locate each variable on its axis, draw a vertical line upward to the ‘Points’ axis to obtain the point value, sum all points, locate the total on the ‘Total Points’ axis, and read the corresponding predicted LNM probability.

Patients were stratified into three risk groups based on tertiles of the combined nomogram score. The observed LNM prevalence increased monotonically from 15.8% in the low-risk group (*n* = 139) to 55.1% in the intermediate-risk group (*n* = 138) and 85.6% in the high-risk group (*n* = 139) (Cochran–Armitage trend *p* < 0.001; Figure 3c). This gradient demonstrates the nomogram’s ability to effectively discriminate patients across the full spectrum of LNM risk. The resulting nomogram, integrating clinical variables with the pathomics score, is presented in Figure 4.

### 3.5. Survival Analysis in the pN0 Subgroup

Follow-up and events. Median follow-up was 49.0 months for overall survival and 49.5 months for progression-free survival (reverse Kaplan–Meier method). Follow-up was administratively censored at the database lock in mid-2019 (last recorded contact 23 July 2019; for censored patients the last recorded contact clustered in May–July 2019). Thirty patients (7.2%) were lost to follow-up before the final year of follow-up, with a median recorded contact of approximately 5 months after surgery. During follow-up, 141 deaths and 160 progression or death events were recorded: 35 and 44 among the 199 node-negative patients, and 106 and 116 among the 217 node-positive patients. Adjuvant chemotherapy was administered to 208 patients (50.0%; 45/199 [22.6%] node-negative and 163/217 [75.1%] node-positive); adjuvant radiotherapy was administered to 19 patients (4.6%), in all cases combined with chemotherapy. Schoenfeld residual testing indicated a departure from the proportional-hazards assumption (OS *p* = 0.011; PFS *p* = 0.032), so the hazard ratios reported below should be read as average effects over the follow-up period rather than as constant instantaneous risks.

Among the 199 node-negative patients, we examined whether the pathomics score could identify a subset at elevated risk of poor survival despite being classified as conventionally ‘low-risk’. Using the subgroup median pathScore as an exploratory, data-driven cutoff, 99 patients were classified as high-risk and 100 as low-risk.

**Figure 5:**
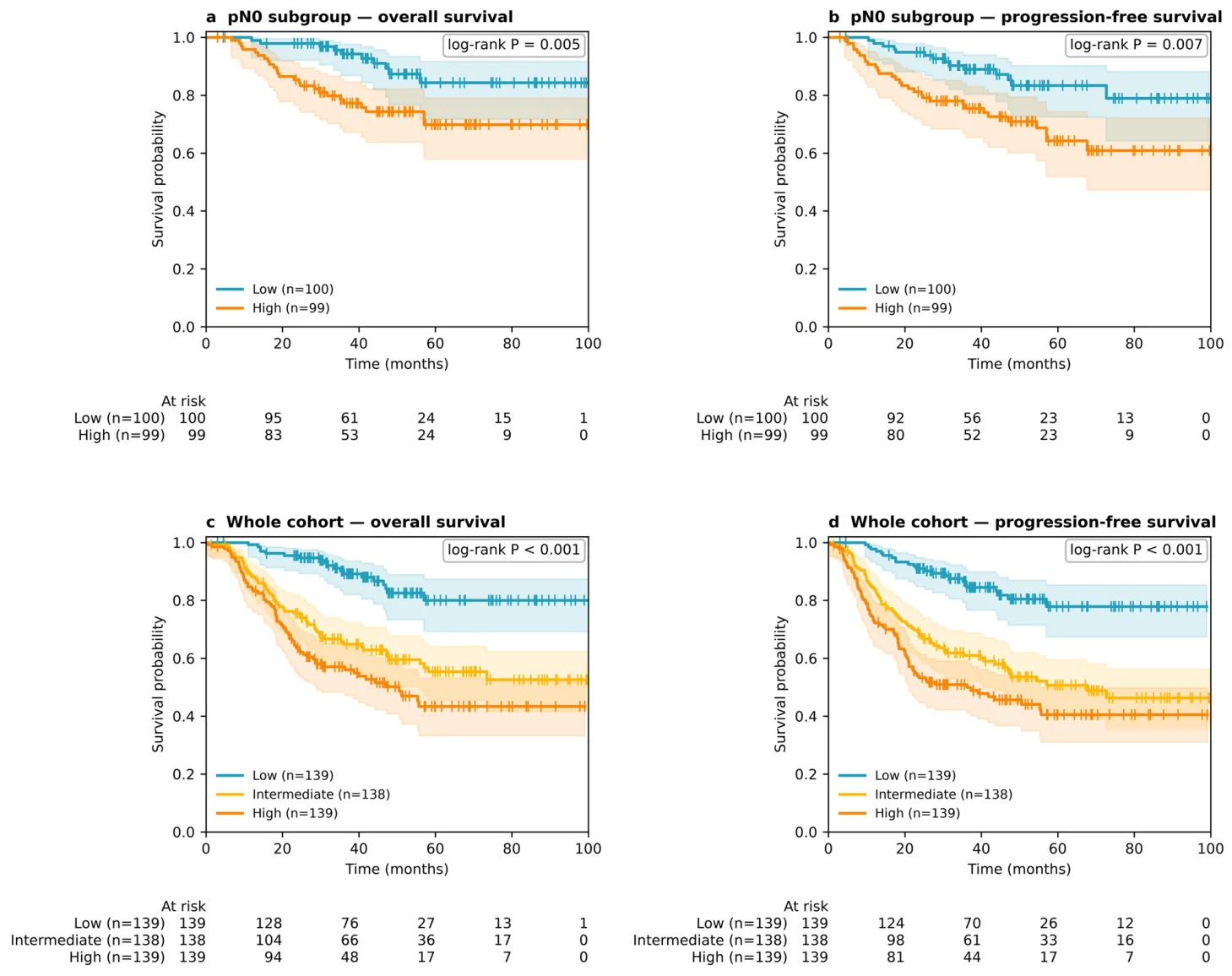
**Kaplan–Meier survival curves.** (**a**,**b**) pN0 subgroup (*n* = 199) stratified by pathomics risk (median pathScore cutoff): (**a**) overall survival (35 events; log-rank *p* = 0.005); (**b**) progression-free survival (44 events; log-rank *p* = 0.007). (**c**,**d**) Whole cohort (*N* = 416) stratified by risk groups defined by the probabilities of the deployed nomogram fitted on the full cohort (exploratory; Section 2.5): (**c**) overall survival (141 events; 3-year OS 89.2%/64.9%/57.1% for low/intermediate/high risk; log-rank *p* < 0.001); (**d**) progression-free survival (160 events; 3-year PFS 85.5%/61.0%/50.9%; log-rank *p* < 0.001). Shaded areas are 95% confidence bands, tick marks denote censored patients, and numbers at risk are given beneath each panel.

High-risk pN0 patients had significantly worse overall survival than their low-risk counterparts (3-year OS: 77.2% vs. 94.3%; log-rank *p* = 0.005; HR per 1-SD increase = 1.49, 95% CI 1.07–2.05; *p* = 0.016). A similar pattern was observed for progression-free survival (3-year PFS: 75.5% vs. 88.9%; log-rank *p* = 0.007; HR = 1.41, 95% CI 1.05–1.88; *p* = 0.020) (Figure 5a,b).

When pathScore was instead entered as a continuous variable and adjusted for age, pathological T category, histological grade, tumor location and adjuvant chemotherapy, the association was attenuated and no longer statistically significant (adjusted HR per 1-SD increase 1.38, 95% CI 0.90–2.12; *p* = 0.142 for overall survival; 1.27, 95% CI 0.87–1.86; *p* = 0.218 for progression-free survival); adjuvant chemotherapy itself was not significantly associated with outcome within the node-negative subgroup (HR 0.66, 95% CI 0.27–1.61, *p* = 0.357 for OS; 0.69, 95% CI 0.31–1.54, *p* = 0.365 for PFS). With 35 and 44 events respectively, these models provided 5.8 and 7.3 events per variable and are therefore underpowered. The continuous analysis is the principal test; the median-dichotomized comparison shown in Figure 5a,b is presented as exploratory.

Sensitivity analysis with extended follow-up. Updating vital status as described above increased median follow-up from 49.0 to 91.0 months and the number of deaths from 141 to 162. Within the node-negative subgroup the unadjusted stratified comparison was, if anything, strengthened (46 events; 3-year OS 76.7% versus 94.8%; log-rank *p* = 1.9 × 10^−4^), whereas the adjusted association of continuous pathScore remained non-significant (adjusted HR 1.23, 95% CI 0.85–1.79; *p* = 0.271). Across the whole cohort the three-year OS gradient was materially unchanged (90.1%, 64.6% and 56.1% in the low-, intermediate- and high-risk groups). These results indicate that the exploratory subgroup finding is not an artifact of the original censoring date, while the adjusted estimate remains underpowered.

In contrast, among pN+ patients (*n* = 217), the pathomics score showed no significant association with OS (log-rank *p* = 0.199), and only a weak association with PFS (log-rank *p* = 0.039). This is consistent with the interpretation that the pathomics signature largely encodes tumor invasiveness and metastatic propensity. This information is already captured once nodal metastasis is established (pN+), so little independent prognostic signal remains within this subgroup; the mechanistic implication is discussed below.

### 3.6. Whole-Cohort Prognostic Stratification

When the entire cohort was stratified by the combined nomogram risk groups, a clear prognostic gradient emerged. For overall survival, the 3-year OS rates were 89.2% (low-risk), 64.9% (intermediate-risk), and 57.1% (high-risk) (log-rank *p* < 0.001; Figure 5c). The corresponding 3-year PFS rates were 85.5%, 61.0%, and 50.9% (log-rank *p* < 0.001; Figure 5d). These findings indicate that the combined model captures biological aggressiveness relevant to nodal status and to long-term survival.

### 3.7. External Validation

**Figure 6:**
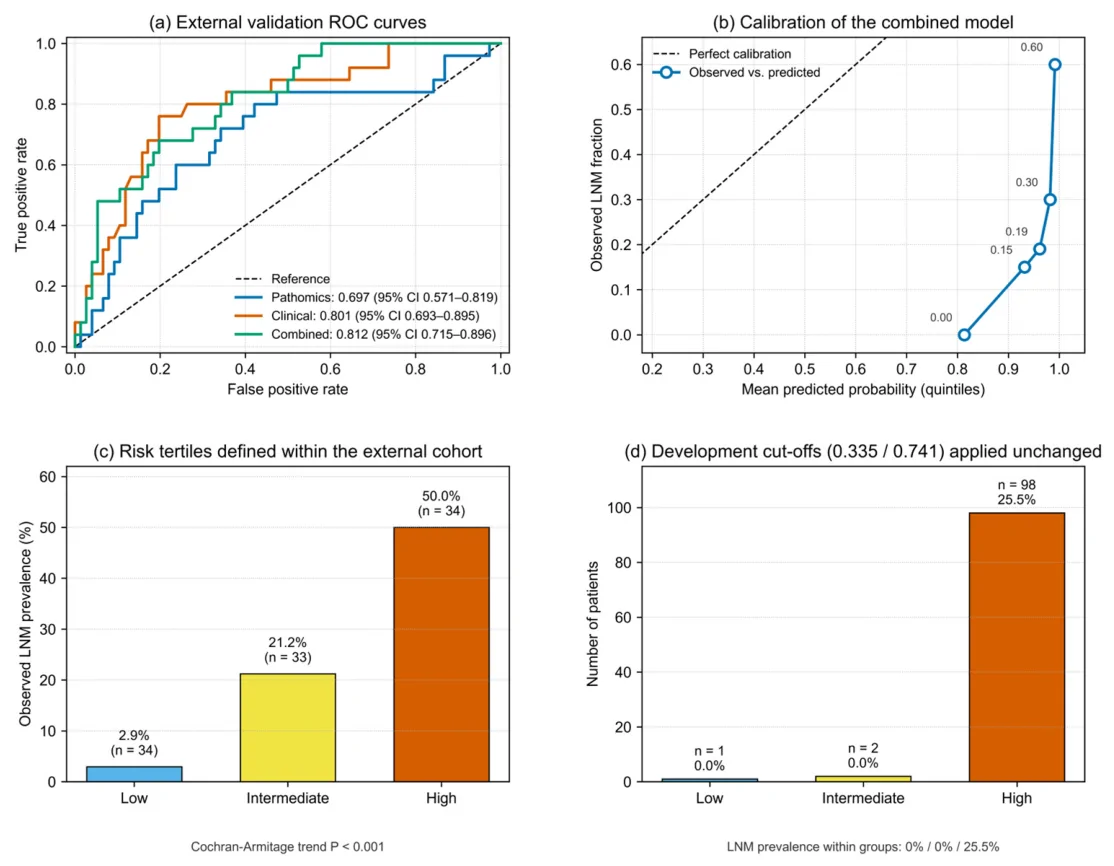
**External validation on an independent cohort of 101 patients.** (**a**) Receiver operating characteristic curves for the three models. (**b**) Calibration of the combined model: observed LNM fractions versus mean predicted probabilities within quintiles of predicted risk; the dashed line indicates perfect calibration. (**c**) Observed lymph-node metastasis prevalence across nomogram risk tertiles defined within the external cohort (*n* = 34, 33, and 34, respectively). (**d**) Risk groups defined by the development-cohort cutoffs (0.335 and 0.741) applied to the external cohort without modification, showing the numbers of patients and the observed LNM prevalence in each group.

To assess transportability, we applied the frozen combined nomogram to an independent external cohort of 101 ESCC patients treated at a separate institution, of whom 25 (24.8%) had pathologically confirmed LNM. The combined model attained an external AUC of 0.812 (95% CI 0.715–0.896), lower than its internal OOF estimate of 0.821 but not significantly different from the external clinical-only model (0.801, 95% CI 0.693–0.895; ΔAUC = +0.011, 95% CI −0.067 to 0.090; DeLong *p* = 0.78; Figure 6a). This difference did not reach statistical significance in a cohort of this size. The pathomics-alone model performed least well externally (AUC 0.697, 95% CI 0.571–0.819; paired bootstrap *p* = 0.021 vs. combined), indicating that the pathomics signature did not generalize to the external center (Table 4). Calibration was poor (slope 1.247, intercept −5.607, Brier 0.648); risk was systematically over-predicted in the external cohort, with predicted probabilities concentrated near the top of the range (median 0.962, interquartile range 0.923–0.984) although only 24.8% of these patients had LNM (Figure 6b). The high Brier score is attributable to this shift alone, because after intercept recalibration, which leaves the ranking of patients unchanged, it falls to 0.145, below the 0.186 obtained by predicting the external prevalence for every patient. The failure is therefore one of calibration, not of ranking. Nevertheless, external risk stratification by nomogram tertiles, defined within the external cohort, remained informative: observed LNM prevalence was 2.9% (low-risk, *n* = 34), 21.2% (intermediate-risk, *n* = 33), and 50.0% (high-risk, *n* = 34) (Cochran–Armitage trend *p* < 0.001; Figure 6c). When the development-cohort cutoffs (0.335 and 0.741) were applied to the external cohort without modification, 1, 2, and 98 patients fell into the low-, intermediate-, and high-risk groups (LNM prevalence 0%, 0%, and 25.5%), because the frozen model systematically over-predicts risk in this cohort; absolute cutoffs derived in the development cohort are therefore not transferable (Table S4).The two cohorts differed in several respects (Table 5): patients in the external cohort were older (63.6 ± 7.2 vs. 58.5 ± 8.3 years), more often had middle-third tumors (81.2% vs. 61.5%), and had a markedly lower prevalence of lymph node metastasis (24.8% vs. 52.2%). Because the frozen model was applied without recalibration, these case-mix differences are a plausible contributor to the lower external discrimination and should be considered when interpreting the estimates. In this external cohort, adding pathomics did not significantly improve discrimination, so external validation did not confirm the incremental predictive value of the pathomics signature. These findings position the present work as a single-center proof of concept: the clinical component transports reasonably, whereas the pathomics component requires multi-center harmonization before it can be relied upon externally.

## 4. Discussion

In this study, we built a postoperative combined clinical-pathomics nomogram to estimate the probability of pathological nodal involvement in a single-center cohort of 416 ESCC patients who underwent radical esophagectomy, and we tested it in an independent external cohort. Three findings stand out. First, the pathomics signature, derived entirely from routine H&E-stained primary tumor slides, carries independent predictive value beyond established clinicopathological factors (adjusted OR 2.47, *p* < 0.001). Second, the combined model showed good discriminative performance (out-of-fold AUC 0.821) with well-behaved calibration and a clear risk gradient across tertiles (LNM prevalence 15.8% → 55.1% → 85.6%). Third, the signature identified a subset of node-negative patients at markedly higher risk of death and recurrence (3-year OS 77.2% vs. 94.3%), which generates, but does not establish, the hypothesis that pathomics could help guide adjuvant-therapy decisions in this conventionally ‘low-risk’ group; whether risk-adapted management of this kind improves outcomes requires prospective testing. We stress that the combined model’s absolute gain over the clinical model was modest (ΔAUC ≈ 0.03); decision curve analysis showed net benefit across most threshold probabilities, yet the clinical value of this increment still needs prospective confirmation. That increment did not reproduce externally: in the independent cohort the combined model did not significantly outperform the clinical model, and the pathomics signature did not generalize adequately. External validation therefore did not confirm the incremental predictive value of pathomics. Because the model is postoperative, it is not a substitute for nodal staging and does not alter the pN category; nodal status is already known when the slide is analyzed. What it adds is a readout from the primary tumor rather than from the nodes, and that readout does not depend on how completely the nodal field was sampled, so it remains informative when the nodal assessment itself is uncertain [4].

The external validation exposes a clear weakness in the pathomics component. Its AUC fell to 0.697, well below the combined model’s 0.812, and the combined model did not significantly outperform the clinical model externally (ΔAUC = +0.011, 95% CI −0.067 to 0.090; DeLong *p* = 0.78). This difference did not reach statistical significance in a cohort of 101 patients with 25 events. Two explanations carry most of the weight, and neither is hardware: scanning and staining were identical at the two centers. The first is a difference in case mix. LNM prevalence was 52.2% in the development cohort but only 24.8% externally, so the external cohort occupies a different region of the predictor–outcome distribution. A signature tuned where prevalence is high loses separation when prevalence roughly halves. This spectrum effect penalizes any model, regardless of how well it is calibrated. The second is pre-analytical handling. The external blocks had been archived at a different institution and underwent routine fixation and processing that differed slightly from ours. Because the selected features quantify hematoxylin and eosin optical density, they report chromatin and cytoplasmic staining intensity. They are therefore more sensitive to such variation than shape-only descriptors would be. The observed pattern fits both: the clinical component, which uses no image intensity, retained its discrimination externally (AUC 0.801), whereas the intensity-dependent pathomic component did not. A third contributor is specific to the aggregation used here. Because the features are standard deviations of per-nucleus measurements, they encode within-case dispersion, which is more sensitive than a mean to how nuclei are detected and segmented. In the external cohort these features shifted upward by 3.37 SD on average, contributing roughly 3.4 logits of the over-prediction, against about 1.2 logits attributable to the lower external prevalence. In short, the signature is useful at our center but cannot be assumed to transfer without recalibration, and ideally harmonization of pre-analytical procedures. That caveat tempers the single-center findings without invalidating them.

The modest absolute ΔAUC needs context. Rank-based AUC alone understates what pathomics adds; metrics that gauge individualized risk estimation tell a clearer story. Adding the pathomics score to the clinical model improved discrimination on every complementary metric: out-of-fold ΔAUC = 0.029 (DeLong *p* = 0.0389), and the integrated discrimination improvement was significant (IDI = 0.0411, 95% CI 0.0137–0.0669; *p* < 0.001). IDI summarizes how far each patient’s estimated probability moves along a continuous scale, which is what a nomogram reports. The net reclassification improvement was not significant (NRI = 0.048, 95% CI −0.039–0.164); NRI depends on arbitrary risk thresholds (we used data-driven tertiles) and is less sensitive than IDI to small, gradual probability shifts, so its null result is not inconsistent with the significant IDI and ΔAUC. Together with the incremental likelihood-ratio test for the pathomics score (χ^2^ = 35.5, *p* = 2.6 × 10^−9^), these results show that pathomics contributes independent signal beyond T stage, grade, and other routine variables. The combined model also beat both single-modality baselines (over the pathomics-only model: ΔAUC = 0.058, *p* < 0.001; over the clinical model: ΔAUC = 0.029, *p* = 0.0389), confirming that pathomics and clinicopathological features are complementary, not redundant. Because the signature comes from one routine H&E slide with no bespoke hardware, it is a low-barrier adjunct that can sharpen individualized LNM risk estimates where current models leave uncertainty, most visibly in the pN0 subgroup where staging is uninformative yet outcomes vary widely.

Practical cost considerations. We deliberately chose the least resource-intensive route to quantitative pathology: the input is a single routine H&E section that is already produced for diagnostic staging, and no additional sectioning, staining, immunohistochemistry, molecular assay, or imaging procedure is required, so there is no added burden on the patient. The incremental laboratory work is therefore limited to whole-slide scanning of one slide per case and a scripted QuPath pipeline whose marginal cost per additional case is low. Against this modest outlay, adding pathScore improved individualized risk estimation beyond the clinical model (ΔAUC = 0.029; IDI = 0.041) and separated prognosis within the pN0 subgroup, in whom conventional staging provides no prognostic discrimination. We do not, however, claim established cost-effectiveness, because no formal health-economic analysis was performed. The decisive question is whether pathomics-guided management changes clinical decisions and improves outcomes at acceptable cost. That question can only be answered prospectively, with the model triggering a predefined clinical action and with costs, downstream testing, and outcomes all measured. We therefore regard these results as supporting a low-barrier adjunct that is ready for prospective evaluation rather than a technology ready for routine use.

Our selected pathomic features are biologically plausible. The LASSO signature concentrates on nuclear shape irregularity (eccentricity, caliper dimensions), hematoxylin OD variability (chromatin texture and nuclear membrane integrity), and eosin OD distribution (cytoplasmic and stromal patterns). These traits encode cellular atypia and architectural disorganization that pathologists have long linked to aggressive tumor behavior [23,24]. The link is not metaphorical. Decades ago, nuclear morphometry of superficial ESCC already showed that nuclear area and shape factor separate tumors with and without nodal metastasis [25]. Across carcinomas, chromatin fractal dimension is an independent prognostic factor that ties architectural complexity to aggressive biology [24]. The ratio of metastatic to examined nodes also refines risk beyond raw counts [26]. Pathomics adds the ability to quantify these patterns objectively and reproducibly across thousands of cells per slide, capturing heterogeneity that subjective grading misses.

Our results fit within and extend the computational-pathology literature on esophageal cancer. Deep learning on H&E slides now predicts treatment response [11], prognosis [12], and molecular alterations [13] in esophageal and gastroesophageal junction cancers; multi-center AI systems improve preoperative nodal diagnosis in ESCC [27]; and multiple-instance learning on whole-slide images predicts nodal metastasis in ESCC [28]. Our single-center, interpretable nuclear-morphometry approach complements these heavier pipelines. Most prior work, however, depends on convolutional networks that need large samples and heavy computation. Our method uses handcrafted nuclear features from widely available software (QuPath) and runs without dedicated GPU infrastructure, making it more accessible and interpretable. That such simple features still yield an OR > 2 for LNM implies a sizable share of metastatic potential resides in basic nuclear phenotypes.

Measured against published CT-radiomics nomograms for ESCC LNM, our out-of-fold AUCs (pathomics-alone 0.762; combined 0.821) sit squarely within the reported range. Tan et al. [29] reached a test AUC of 0.773 for a radiomics nomogram in resectable ESCC, showing that H&E-derived nuclear features match cross-sectional radiomics without dedicated imaging. Newer CT-radiomics studies report higher values, but cohort and stage differences block direct comparison. Against H&E-based deep learning, Ren et al. [30] reported an internal-test AUC of 0.949 for LNM in T1 ESCC from whole-slide images, yet that work studied a narrower, earlier-stage (endoscopic-resection) population where the task is easier; the higher AUC reflects both the network and the homogeneous low-stage cohort, not a head-to-head win over routine nuclear morphometry. Taken together, single-section pathomic features give LNM discrimination on par with radiomics and form a practical, low-cost complement to clinical staging, though external validation is still required before any superiority claim.

The pN0 subgroup deserves particular attention. Guidelines recommend observation, not adjuvant therapy, for completely resected node-negative ESCC, yet up to one-third of these patients still recur [31]. Our data generate the hypothesis that primary-tumor pathomic analysis could flag pN0 patients at high enough risk to warrant closer surveillance or adjuvant treatment; this remains untested, and we do not claim that pathomics-guided management improves outcomes. If confirmed prospectively, this approach could refine management without extra invasive procedures or molecular assays.

One boundary condition on pathScore’s prognostic value deserves emphasis. Within the pN+ subgroup, pathScore did not predict survival, yet it sharply stratified the pN0 subgroup. The simplest explanation is that pathScore largely encodes tumor invasiveness and metastatic propensity, information already captured once nodal metastasis is present, so little independent prognostic signal remains in pN+ patients. PathScore’s intended use is estimating the probability of nodal involvement from the resection specimen, so this pattern does not undermine that use; it suggests that pathScore and pN status reflect the same underlying biology rather than independent signals. A natural next step is to test whether pathScore keeps independent prognostic value in external pN+ cohorts enriched for covariates such as metastatic-node count, adjuvant treatment, and molecular subtype, and whether it predicts non-survival endpoints like adjuvant-therapy response.

This study has several limitations. First, the external cohort was small (*n* = 101; events = 25), and the combined model’s external AUC (0.812) remained below its internal OOF estimate (0.821) and did not significantly exceed the external clinical model (0.801; ΔAUC = +0.011, *p* = 0.78)(Table 5). The pathomics signature in particular transported poorly (external AUC 0.697). This probably reflects both a spectrum effect (LNM prevalence 24.8% externally versus 52.2% internally) and differences in pre-analytical tissue fixation and processing between the two archival series. Multi-center validation with prospective harmonization of pre-analytical procedures is still needed to prove generalizability. In addition, because the model was applied externally as a frozen model without recalibration, the cutoffs defining the risk groups were derived within each cohort; the nomogram-predicted probabilities are therefore best interpreted as a within-cohort ranking of risk rather than as absolute risks that transfer unchanged between centers. Accordingly, when the development-cohort cutoffs were applied externally without modification, 98 of 101 patients fell into the highest group, and the Brier score of the frozen model (0.648) was worse than that of a prevalence-only prediction (0.186), although it fell to 0.145 after intercept recalibration. Second, the retrospective design carries selection bias, and excluding mostly incomplete-data patients may have enriched the cohort toward earlier, better-documented cases. Third, features came from one representative section per patient, which may miss intratumoral heterogeneity; calibration (Brier score, calibration plot, intercept, and slope in Figure 3a) was generally good, but these metrics still need external validation. Fourth, no tumor region of interest was delineated, and non-neoplastic elements within the analyzed area, including stromal and inflammatory cells, were included in feature computation; the pathomics signature therefore characterizes the composition of the tumor-bearing section rather than tumor nuclei alone, so that part of the signal it captures may reflect stromal or inflammatory response rather than tumor-intrinsic biology. Because such compositional features are sensitive to fixation, processing, and section selection, this choice is a plausible additional contributor to the limited external transportability of the pathomics signature; it does not, however, reflect differences in region-of-interest definition between the two centers, because no region of interest was delineated at either. Fully automated tumor segmentation with tumor-nucleus-specific feature extraction should be explored. Slide selection was performed by one pathologist and reviewed by a second, both blinded to lymph node status and survival outcomes, but no formal reproducibility statistics for slide selection were calculated. Fifth, whether the modest incremental gain over the clinical model (ΔAUC ≈ 0.03) justifies the cost of slide digitization and feature extraction remains to be tested in prospective trials that ask whether pathomics-guided decisions improve outcomes. Sixth, by restricting the cohort to patients who underwent upfront surgery, the model applies to treatment-naive ESCC; because neoadjuvant or perioperative therapy has become standard for locally advanced disease in many centers, a biopsy-derived pathomic model will be needed before this approach can be extended to that setting. The 2012–2017 treatment era itself also limits applicability to contemporary practice, in which staging and perioperative management have evolved.

Even with these limitations, this study shows that quantitative pathomic analysis of routine H&E slides provides a modest but consistent increment to standard clinicopathological assessment for estimating the probability of pathological nodal involvement in ESCC from the resection specimen. Clinically, the pathomic-augmented nomogram singles out node-negative patients at elevated risk of poor outcomes, a subgroup that current staging cannot flag. If confirmed in prospective studies, this could support a targeted-surveillance role that complements rather than replaces pathology review. Because the pathomic signature transported poorly across centers whereas the clinical component did not, harmonization of pre-analytical procedures and center-specific recalibration are the rate-limiting steps before deployment. Future work should pursue deep-learning feature extraction for possible performance gains, test this targeted-surveillance strategy prospectively in high-risk node-negative patients, and establish standardized pre-analytical protocols that let the signature travel.

## 5. Conclusions

We developed and internally validated a postoperative pathomics–clinical nomogram that estimates the probability of pathological lymph node metastasis in ESCC from the resection specimen. The pathomics signature, derived from routine H&E slides via QuPath-based nuclear morphometric analysis, is an independent predictor of nodal metastasis (OR 2.47, *p* < 0.001) and identifies high-risk patients within the conventionally node-negative population. These findings support the potential role of pathomics as a low-barrier complement to current staging systems for refined postoperative risk stratification in ESCC. External validation in an independent cohort did not confirm the incremental predictive value of pathomics, and the combined model did not significantly outperform the clinical model there; these results should therefore be read as proof of concept at the development center rather than as a transportable pathomics–clinical model. Multi-center prospective validation is warranted to confirm generalizability and assess clinical impact.

## Figures and Tables

**Table 1:** Baseline characteristics of the study cohort.

Variable	Overall (*N* = 416)	pN0 (*n* = 199)	pN+ (*n* = 217)
Age, years, mean ± SD	58.5 ± 8.3	58.8 ± 8.3	58.2 ± 8.4
Sex, male, n (%)	306 (73.6%)	133 (66.8%)	173 (79.7%)
Tumor location, n (%)			
Upper	34 (8.2%)	17 (8.5%)	17 (7.8%)
Middle	256 (61.5%)	129 (64.8%)	127 (58.5%)
Lower	126 (30.3%)	53 (26.6%)	73 (33.6%)
Histological grade, n (%)			
G1	174 (41.8%)	92 (46.2%)	82 (37.8%)
G2	213 (51.2%)	97 (48.7%)	116 (53.5%)
G3	29 (7.0%)	10 (5.0%)	19 (8.8%)
Pathological T category (pT1/pT2 vs. pT3/pT4), n (%)			
pT1/pT2	210 (50.5%)	152 (76.4%)	58 (26.7%)
pT3/pT4	206 (49.5%)	47 (23.6%)	159 (73.3%)
Lymph nodes retrieved, mean ± SD	32.0 ± 12.8	31.0 ± 12.9	33.0 ± 12.6

**Table 2:** Multivariable logistic regression for LNM prediction (combined model, *N* = 416). Incremental likelihood-ratio test for pathomics score: χ^2^ = 35.5, *p* = 2.56 × 10^−9^.

Variable	OR	95% CI	*p*-Value
Sex	1.553	0.870–2.772	0.138
Age, years	0.993	0.964–1.023	0.639
Tumor location	1.454	0.949–2.230	0.086
Pathological T category (pT)	2.617	1.904–3.598	3.56 × 10^−9^
Grade	1.681	1.120–2.524	0.013
Pathomics score	2.470	1.801–3.387	1.97 × 10^−8^

**Table 3:** Internal validation performance of the three models (*N* = 416). OOF = out-of-fold (10-fold cross-validation); apparent = resubstitution; optimism = apparent − OOF. DeLong P compares each model against the combined model. ΔAUC values are computed as combined model minus comparator, so that positive values favor the combined model; 95% confidence intervals were obtained by paired bootstrap resampling (2000 replicates) of the out-of-fold predictions. ΔAUC values are computed from unrounded AUC estimates and may differ by 0.001 from differences of the rounded values shown in the table.

Model	OOF AUC (95% CI)	Apparent AUC	Optimism	*p* vs. Combined (DeLong)	ΔAUC (Combined−Model) (95% CI)
Clinical	0.791 (0.744–0.835)	0.803	+0.012	0.0389	0.029 (0.003 to 0.055)
Pathomics	0.762 (0.715–0.806)	0.795	+0.033	0.0006	0.058 (0.025 to 0.092)
Combined	0.821 (0.779–0.858)	0.847	+0.026	reference	(reference)

**Table 4:** External validation performance of the three models (independent cohort, *N* = 101). AUCs with 95% CIs from 2000 bootstrap resamples. Brier score and calibration slope/intercept are reported for the combined model; the Brier score is also given after intercept recalibration (0.145), an explanatory analysis that separates failure of calibration from failure of ranking. The pathomics and clinical models are shown for discrimination comparison. *p*-values compare each model with the combined model: DeLong’s test for the clinical model, and paired bootstrap resampling (5000 replicates) for the pathomics model, which is reported because the external predicted probabilities were highly concentrated and the models are nested, conditions under which the DeLong covariance estimator is unstable (Section 2.4).

Model	External AUC (95% CI)	*p* vs. Combined	Brier	Calibration Slope	Calibration Intercept
Clinical	0.801 (0.693–0.895)	0.78	—	—	—
Pathomics	0.697 (0.571–0.819)	0.021	—	—	—
Combined	0.812 (0.715–0.896)	reference	0.648	1.247	−5.607

**Table 5:** Baseline characteristics of the development and external validation cohorts. Values are n (%) unless stated otherwise.

Characteristic	Development Cohort (*N* = 416)	External Cohort (*n* = 101)
Age, years, mean ± SD	58.5 ± 8.3	63.6 ± 7.2
Sex, male, n (%)	306 (73.6%)	67 (66.3%)
Tumor location, upper, n (%)	34 (8.2%)	3 (3.0%)
Tumor location, middle, n (%)	256 (61.5%)	82 (81.2%)
Tumor location, lower, n (%)	126 (30.3%)	16 (15.8%)
Pathological T category, pT1/pT2, n (%)	210 (50.5%)	52 (51.5%)
Pathological T category, pT3/pT4, n (%)	206 (49.5%)	49 (48.5%)
Histological grade, G1, n (%)	174 (41.8%)	31 (30.7%)
Histological grade, G2, n (%)	213 (51.2%)	58 (57.4%)
Histological grade, G3, n (%)	29 (7.0%)	12 (11.9%)
Lymph node metastasis, n (%)	217 (52.2%)	25 (24.8%)

## Data Availability

The anonymized clinicopathological dataset and the extracted pathomic feature matrix supporting the findings of this study are available from the corresponding author upon reasonable request, subject to institutional data-sharing approval. The whole-slide images cannot be made publicly available owing to patient-privacy restrictions. The QuPath scripts used for nucleus detection and feature extraction, together with the frozen model specification (StandardScaler constants, LASSO-selected pathomic feature weights and logistic regression coefficients, tabulated in Table S1), are provided as Supplementary Materials, so that the published model can be applied to new slides without access to the development data. Patient-level data are not released.

## Supplementary Materials

The following supporting information can be downloaded at https://www.mdpi.com/article/10.3390/cancers18203248/s1: Script S1. QuPath pipeline for nucleus detection and 41-feature extraction; Table S1. Frozen model specification (StandardScaler constants, LASSO-selected pathomic feature weights and logistic regression coefficients); Table S2. Completed TRIPOD+AI reporting checklist; Table S3. Completed CLAIM 2024 reporting checklist; Table S4. Sensitivity of the external validation to the feature-aggregation function (development and external cohorts analyzed with standard-deviation versus mean aggregation). Table S5. Sensitivity of the internal bootstrap confidence intervals to repetition of the complete model-development process (2000 replicates), with the distribution of the number of LASSO-selected features.

## Abbreviations

The following abbreviations are used in this manuscript:

AJCC	American Joint Committee on Cancer
AUC	Area under the receiver operating characteristic curve
CI	Confidence interval
CT	Computed tomography
DCA	Decision curve analysis
ESCC	Esophageal squamous cell carcinoma
EUS	Endoscopic ultrasonography
FFPE	Formalin-fixed paraffin-embedded
H&E	Hematoxylin and eosin
HR	Hazard ratio
KM	Kaplan–Meier
LASSO	Least Absolute Shrinkage and Selection Operator
LNM	Lymph node metastasis
OD	Optical density
OR	Odds ratio
OS	Overall survival
PET	Positron emission tomography
PFS	Progression-free survival
pN0	Pathologically node-negative
pN+	Pathologically node-positive
SD	Standard deviation
TNM	Tumor–node–metastasis
UICC	Union for International Cancer Control
WSI	Whole-slide image
